# Teledermatology in remote Indigenous populations: Lessons learned and paths to explore, an experience from Canada (Québec) and Australia

**DOI:** 10.1177/20552076231217813

**Published:** 2023-11-29

**Authors:** Alex Nguyen, Catherine K Zhu, Elizabeth O’Brien

**Affiliations:** 1 12367 Faculty of Medicine, McGill University, Montréal, Quebec, Canada; 2 Division of Dermatology, 12367 McGill University Health Center, Montréal, Quebec, Canada

**Keywords:** Teledermatology, telemedicine, indigenous, rural, healthcare, canada, quebec, australia, challenges, narrative review

## Abstract

**Objective:**

Recent introduction of a provincially funded and administered teledermatology platform in Quebec presents a major opportunity to improve healthcare delivery to rural Indigenous communities where healthcare is suboptimal. In this study, we assessed approaches, challenges, solutions, and outcomes in implementing teledermatology in rural Indigenous communities of Australia and Canada.

**Methods:**

A narrative review was performed using journal articles and grey literatures to assess challenges encountered in Canadian and Australian teledermatology programs in rural Indigenous communities. We then conducted a focused search to identify solutions and outcomes to these challenges. We identified four main areas of focus for implementing teledermatology: financial, cultural, legal, and provider competency.

**Results:**

Main financial concerns included identifying the cost-to-benefit ratio of teledermatology and financial benefits of the store-and-forward system compared to videoconferencing. Delivery of teledermatology through culturally considerate services is crucial to mend the mistrust felt by Indigenous people toward mainstream health services. From a legal standpoint, patient confidentiality and physician liability must be considered. A uniform teledermatology platform and physician competency in both telemedicine and dermatology are needed to ensure standard of care.

**Conclusion:**

Teledermatology initiatives represent great opportunities to improve healthcare services to rural Indigenous populations.

## Introduction

“Indigenous” refers to native inhabitants of a region with distinct cultural, traditional, and social practices. They often experience health disparities and poorer health care outcomes due to reduced healthcare access.^1,2^ Teledermatology, a branch of telemedicine, has gained popularity over the years due to cost-effectiveness and convenience. On July 4, 2022, Québec launched its own teledermatology program, following Australia's footsteps as the pioneers in telemedicine.^3^ The program, led by the *Minister of Health and Social Services*, *the Réseau Québécois de la Télésanté*, and the *Association des médecins spécialistes dermatologues du Québec*, aims to improve access to care for the general population and the quality of care for rural Indigenous communities.

**Figure 1.**
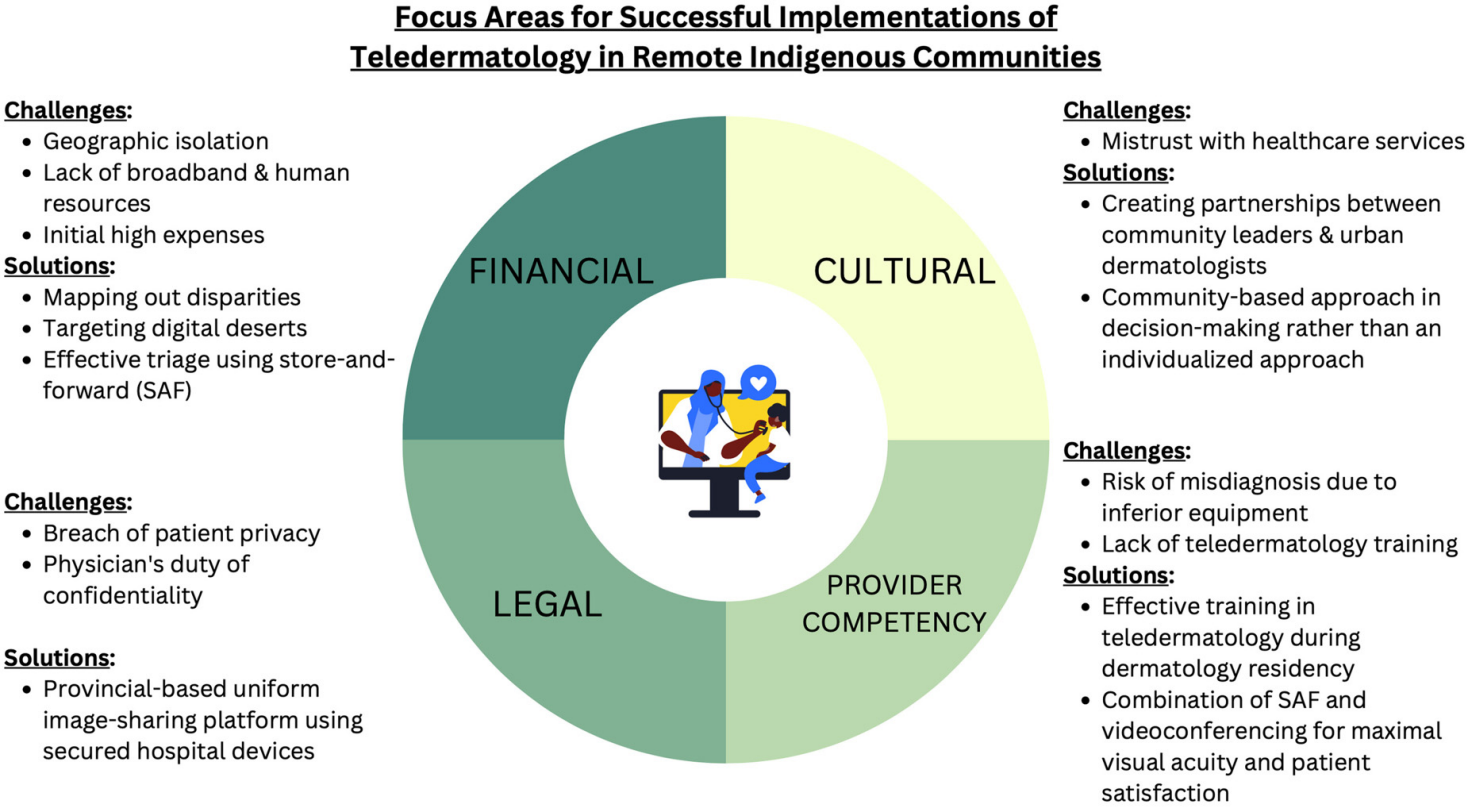
Focus areas for successful implementations of teledermatology in remote Indigenous communities.

The launch of a new teledermatology program is bound to face challenges. Canada can learn from Australia's experiences as they share similarities in their large remote Indigenous communities, healthcare delivery structure, and economic and political status.^4^ Both countries face barriers such as financial constraints, cultural differences, legal considerations, and provider competency. By analyzing Australia's success and challenges, an effective plan of action to optimize teledermatology can be developed for remote Indigenous communities in Québec and the rest of Canada. In this narrative review, we explore the shared barriers in teledermatology delivery and review the solutions and outcomes of each (summarized in Figure 1).

## What are the financial considerations for implementing teledermatology in remote Indigenous communities?

Financial considerations are critical in successfully implementing teledermatology in remote Indigenous communities. The unique challenges faced by these populations, such as geographic isolation, lack of broadband, and human resources, can hinder access to teledermatology programs.

For isolated remote Indigenous populations, the initial costs of providing equipment and resources due to inconsistent road access can be substantial. However, data from the early 2000s demonstrate that despite the initial transportation costs, teledermatology can lead to significant long-term savings by reducing unnecessary travel expenses for patients in the future.^5^ These travel expenses can critically impede access to dermatological care.

Further, an Australian study on teledermatology in 2019 has shown that targeting digital deserts and bridging the technology gap can yield financial gains in the future.^6^ Moreover, improved broadband connectivity not only enhances dermatology services in rural areas but also offers better medical education opportunities for medical students and residents, making it an attractive incentive for financing teledermatology programs despite their initial expenses.^7^

A scoping review analyzing data from 1993 to 2020 revealed that videoconference appointments for teledermatology were initially more costly than in-person appointments.^8^) However, a 2016 systematic review found that the store-and-forward system, allowing dermatologists to review images and information at their convenience, led to a reduction of in-person appointments, thus cutting travel costs and overall teledermatology expenses.^9^ This cost–benefit ratio was especially favorable for patients residing far from in-person dermatology services.^10^

To ensure the financial viability of a government-funded teledermatology program in Québec, investments in digital infrastructure should be prioritized to generate long-term cost savings, improve rural medical education, and implement the store-and-forward strategy for teledermatology.

## How can the mistrust of the Indigenous communities be addressed in the implementation of a teledermatology program?

When introducing Québec's teledermatology program for Indigenous communities, it is crucial to address the deep-rooted mistrust that Indigenous people may harbor due to historical and systemic trauma. Throughout history, Indigenous people of Québec have endured colonization, oppression, and resource inequalities, resulting in a lack of trust in mainstream healthcare systems. In fact, an Australian study showed that most Indigenous people avoid seeking care from public health services due to fears of racism, disrespect, and negative interactions with healthcare staff.^11^ Furthermore, a 2022 Canadian review suggests that Indigenous parents often fear seeking healthcare because of the risk of having their children apprehended by hospital staff.^12^

To alleviate these fears and promote trust, it is essential to involve Indigenous healthcare practitioners in the development and implementation of the teledermatology program. Collaboration between public health and Indigenous communities can lead to the creation of culturally competent and safe healthcare services. Australian reviews in 2021 have suggested that partnerships with community Elders and family groups, along with the employment and retention of Indigenous staff, can contribute to culturally appropriate care.^11,13^ For example, the Australian government established a partnership between mainstream health services and Aboriginal community-controlled health services (ACCHS). This model allows Indigenous patients to attend videoconferences at ACCHS and consult with specialists located in public hospitals.^14^ The presence of an Indigenous healthcare worker during telehealth consultations also enhances the understanding of patients’ personal circumstances, health needs, and behaviors toward medical specialists.^15^

A Canadian critical review data in 2017 suggest that Indigenous populations have different approaches to health, prioritizing community-based decision-making and holistic well-being. For instance, an Australian study looking at the management of diabetes through telemedicine found that the most appreciated aspect of the program by Indigenous users was the culturally specific peer support that fostered a feeling of safety.^16^ The ideal teledermatology strategy should therefore adopt a comprehensive model of healthcare that addresses all aspects of health, including social and emotional well-being, in one visit.^17^

Québec needs to establish a teledermatology program that integrates Indigenous viewpoints, cultural competence, and community collaboration to overcome healthcare barriers and enhance Indigenous access to quality care.

## How can issues of patient privacy, confidentiality, and physician liability be addressed in teledermatology programs?

The successful management of any telemedicine program requires special consideration of patient privacy, confidentiality, and physician liability. The issues of patient privacy and confidentiality are even more important in teledermatology depends on sharing digital photographs of skin lesions. Unfortunately, with the sharing of clinical photography, a 2018 Australian systematic review marked that breach of patient privacy may occur with the use of personal smartphones and devices to capture these pictures.^9^ To prevent privacy breaches, a province-based uniform platform for picture sharing using hospital devices should be established.

Physician liability may also cause issues in a teledermatology program. A 2020 systematic review on the diagnosis and management of melanoma showed that there is a risk of incorrect diagnosis due to inferior technology and not being able to perform a full body exam by a dermatologist.^18^ To mitigate these risks, updated Australian teledermatology guidelines in 2020 recommended ensuring patient indemnity insurance covers telehealth, obtaining informed consent, and providing training in dermoscopy for remote Indigenous community physicians.^18,19^

By addressing these important considerations, teledermatology can provide quality care while protecting patient privacy, ensuring proper funding, and minimizing physician liability risks.

## How can healthcare providers improve their competency in teledermatology?

Providing dermatological care through virtual consults poses unique challenges for dermatologists, including diagnosis, treatment, and follow-up. A limitation of teledermatology identified in a 2013 Australian audit is the lack of information on patient outcomes following treatment plans.^20^ To address this, a proposed solution from a 2021 qualitative study adopted the concept of an expanded role of nursing where nurses in remote Indigenous communities have a skillset to diagnose, treat, and follow up on certain dermatological conditions.^21^

Virtual consults also increase the risk of misdiagnosis due to the use of inferior technology and the inability to conduct full-body skin examinations. Educating community nurses and physicians on full-body skin exams would help mitigate this issue. Given that dermoscopy has been identified as a crucial diagnostic tool, studies on teledermoscopy suggested that training Indigenous community nurses and family physicians in dermoscopy can improve diagnostic accuracy.^22,23^ Virtual dermatoscope images could be sent by store-and-forward system to the dermatologist to be assessed.^22^ The teledermoscope could be used to monitor doctor-identified skin lesions to help with patient follow-up as suggested by an Australian study.^23^

Furthermore, a study performed by the *Australian College of Dermatologists* recognized the store-and-forward system as the most efficient delivery method of teledermatology, providing high-quality images for evaluation.^24^ A 2005 study suggested that while videoconference alone may not enhance diagnostic accuracy significantly, combining it with store-and-forward can improve patient satisfaction and trust.^25^

It is essential to adapt the training of dermatologists for the successful implementation of teledermatology. This adaptation should focus on developing expertise in dermatoscope image interpretation, an understanding of the limitations of teledermatology, and the knowledge to select appropriate patients for virtual.^19^

## Conclusion

Teledermatology initiatives hold tremendous significance for improving healthcare access in rural Indigenous populations across Canada. Thus, it remains imperative to thoroughly evaluate potential challenges spanning financial, cultural, legal, and competency realms that could impede the implementation and long-term sustainability of teledermatology in remote Indigenous communities. Québec and other Canadian regions can derive invaluable insights from Australia's proactive approach to overcoming similar barriers to ensure successful healthcare access for Indigenous populations.
